# A Comparison of Delayed Self-Heterodyne Interference Measurement of Laser Linewidth Using Mach-Zehnder and Michelson Interferometers

**DOI:** 10.3390/s111009233

**Published:** 2011-09-27

**Authors:** Albert Canagasabey, Andrew Michie, John Canning, John Holdsworth, Simon Fleming, Hsiao-Chuan Wang, Mattias L. Åslund

**Affiliations:** 1 Interdisciplinary Photonics Laboratories (iPL), School of Chemistry, University of Sydney, 2006, NSW, Australia; E-Mails: andrew.michie@sydney.edu.au (A.M.); john.canning@sydney.edu.au (J.C.); soongking@gmail.com (H.-C.W.); mattias.aslund@gmail.com (M.L.Å.); 2 Institute of Photonics and Optical Science (IPOS), School of Physics, University of Sydney, 2006, NSW, Australia; E-Mail: simon.fleming@sydney.edu.au (S.F.); 3 SMAPS, University of Newcastle, Callaghan, NSW 2308, Australia; E-Mail: john.holdsworth@newcastle.edu.au (J.H.)

**Keywords:** coherent optical effects, heterodyne, interferometry, DFB, laser linewidth, faraday rotation, Mach-Zehnder interferometer, Michelson interferometer, Gaussian noise, Lorentzian noise

## Abstract

Linewidth measurements of a distributed feedback (DFB) fibre laser are made using delayed self heterodyne interferometry (DHSI) with both Mach-Zehnder and Michelson interferometer configurations. Voigt fitting is used to extract and compare the Lorentzian and Gaussian linewidths and associated sources of noise. The respective measurements are *w_L_* (MZI) = (1.6 ± 0.2) kHz and *w_L_* (MI) = (1.4 ± 0.1) kHz. The Michelson with Faraday rotator mirrors gives a slightly narrower linewidth with significantly reduced error. This is explained by the unscrambling of polarisation drift using the Faraday rotator mirrors, confirmed by comparing with non-rotating standard gold coated fibre end mirrors.

## 1. Introduction

The inherently narrow linewidths of distributed feedback (DFB) fiber lasers make them particularly attractive for applications such as optical communications, sensing and spectroscopy. The application of the Schawlow-Townes formula to DFB fiber lasers indicates that the linewidth can be 60 Hz or less [1]. However, in practice, the observed linewidths have been significantly greater, owing to increased phase noise, which is often attributed to environmental perturbations. Delayed self-heterodyne interferometery (DSHI) has been employed for the measurement of laser linewidths since it was introduced in 1980 [2]. It has been particularly useful for the measurement of linewidths in semiconductor DFB lasers for which the most significant contribution to noise arises from spontaneous emission-induced refractive index changes [3]. This noise is predominantly white in nature, for which the lineshape is Lorentzian. For DFB fiber lasers, however, the linewidth can be greatly influenced by other sources of noise, which tend to be colored in nature and characterised with a Gaussian lineshape [4,5]. The presence of both sources of noise with dissimilar lineshapes complicates the analysis for the extraction of linewidth for the DFB fiber laser. A convolution of the Lorentzian and Gaussian functions, such as the Voigt function, is typically required for linewidth extraction from interferometric measurements of fibre DFB laser outputs [6].

In practice, the contribution to the Gaussian linewidth has been primarily attributed to thermal noise in the DFB cavity leading to refractive index fluctuations [4,5]. The main contributors to these thermal fluctuations have been identified to be ambient temperature fluctuations, self-heating, and fundamental temperature noise due to random diffusion of phonon-phonon coupled excitations [5] with acoustic fluctuations also being a potential contributor. Furthermore, self-heating of the DFB as a result of non-radiative dissipated pump power has also been identified as an additional source of potential noise capable of further broadening the linewidth [7,8]. An increase in linewidth, for both Lorentzian and Gaussian contributions, has been observed with increasing pump powers [8], attributed to temperature fluctuations caused by variations in absorbed pump energy. This apparent contravention of the Schawlow-Townes formula for the increase in the Lorentzian linewidth was argued as evidence that that the broadening may not be entirely related to spontaneous emission alone [9]. The transition from the low pump power regime where the noise predominantly derives from intrinsic thermal fluctuations to the high power regime where pump noise becomes significant has also been observed in a direct measurement of the frequency noise of the DFB laser [10]. Hence, there are a number of contributions to noise both within the laser itself and from external sources including the pumping system, which cannot always be well quantified but can be qualitatively determined within the two profiles used to extract linewidth.

The direct measurement of linewidth using DSHI is typically carried out using a Mach-Zehnder interferometric (MZI) configuration employing conventional low-birefringence fiber. The MZI is prone to signal degradation as a result of fringe fading caused by random variations in the state of polarization (SOP) of the two arms with respect to each other. This contribution is generally more problematic with increasing interferometer path length (necessary with decreasing laser linewidth) and can potentially cause signal (fringe) fading and result in significant measurement errors in the laser linewidth. The use of expensive high-birefringence fiber and components based on such fiber is typically ruled out given the very long delay lengths required for the DSHI and the associated complexities. Instead, a polarization controller is inserted into one of the arms to match the SOP at the beginning of each measurement. Unfortunately, this is not ideal given the long measurement times required for the acquisition of the beat spectrum over which time the SOP will drift due to environmental perturbations no matter how shielded the setup. This drift in polarization affects the observable interference and raises questions about the inherent noise in the MZI measurements. From an interferometery perspective, a solution is to use a Michelson interferometer (MI) which employs Faraday rotator mirrors (FRM) to compensate for birefringence effects, providing a polarization-insensitive measurement technique that is immune to fringe fading [11]. This MI configuration with Faraday mirrors has been used previously to reduce laser linewidth through active electronic feedback [12].

In this paper, we explore the potential for using this type of MI configuration to measure laser linewidths, in particular DFB fibre lasers. A Voigt fit is used to extract the Gaussian and Lorentzian components of the linewidths of a DFB fiber laser in both these interferometric configurations for varying power levels. A comparison is made between interferometers in the context of the extracted linewidths. In particular, we examine whether or not the MI offers any improvements over the MZI.

## 2. Experiments

### 2.1. DFB Characterization

**Figure 1.**
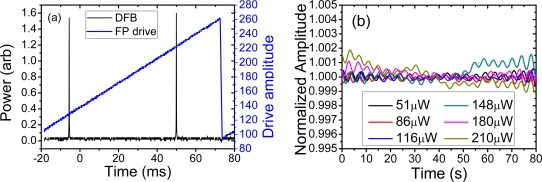
(**a**) Longitudinal mode structure of DFB acquired using a scanning Fabry-Perot (FP) and an oscilloscope; (**b**) Amplitude fluctuations in DFB for varying power measured directly on an oscilloscope.

The DFB laser used in these experiments was fabricated by direct writing with a continuous-wave 244 nm from a frequency doubled Ar^+^ laser into an Er^3+^ doped germanosilicate fiber. The phase-shifted grating is 55 mm long with an off-centre phase shift designed to preferentially direct single polarisation laser output from one side. The laser was mounted on an aluminum block and covered in heat conductive paste. It was pumped with a 976 nm grating stabilized laser diode, and the DFB fibre laser output wavelength measured to be *λ* ∼ 1,547 nm. The longitudinal mode profile was characterized using a scanning Fabry-Perot (FP), shown in Figure 1(a)—this confirmed that the laser was robustly operating on one linear polarization eigenstate. In order to estimate the degree of intrinsic laser signal variation, the amplitude variations in the DFB output were measured on an oscilloscope and are shown for varying power levels in Figure 1(b). For the highest output power used, the variation in the power of the DFB was measured to be <0.2% indicating a well fabricated, stable single polarization longitudinal mode DFB laser, an ideal source for comparing the interferometers.

### 2.2. Interferometers

**Figure 2.**
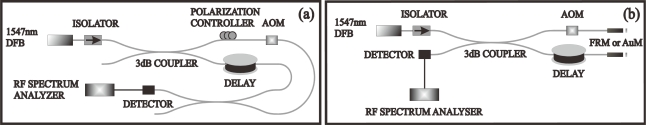
Delayed self-heterodyne interferometer (DHSI) technique using (**a**) a Mach-Zehnder interferometer (MZI); and (**b**) a Michelson interferometer (MI) with FRMs. (AOM: Acousto-Optic Modulator, FRM: Faraday Rotator Mirror, RF: Radio Frequency).

For the DSHI experiments, a MZI and a MI employing FRMs were constructed as shown in Figure 2. The DFB laser and the interferometers were placed inside a soundproof enclosure which was placed on an actively isolated electronic vibration isolation table.

In the MZI configuration shown in Figure 2(a), the light from the DFB laser is split using a 2 × 2 coupler into two arms, with light in one arm experiencing a 42 km delay while in the other arm an acousto-optic modulator (AOM) is used to shift the frequency of light by *f* = 27.12 MHz. Light recombines at a second 2 × 2 coupler with the interference signal detected by a fast photodiode. A polarization controller is used to optimize the fringe visibility of the MZI.

In the Michelson configuration (Figure 2(b)), the second 2 × 2 coupler is removed and the two arms are terminated with either FRMs or straight gold coated mirrored fibre ends. The FRM is expected to compensate for arbitrary rotations in the polarization eigenstates in the two arms and the two beams recombine at the 2 × 2 coupler at the same polarization state as each other but rotated 90° from the initial state of polarization [11]. The delay coil used in the MI configuration is *l* = 21 km, which gives an effective length of *l* = 42 km for the MI, comparable to the delay length of the MZI. The frequency shift in the other arm in this case is also doubled to *f* = 54.24 MHz with the return trip.

The beat spectra resulting from the interference is viewed in both configurations using a radio-frequency spectrum analyzer (RFSA—Agilent N9320B) and an approximate value for the DFB coherence length can be simply determined from the RFSA trace. When the MZI delay length, *l* = 42 km, the recombining light is no longer interfering and therefore incoherent—*i.e.*, there is no observable interference signal and/or delta peak. At a MZI delay length of *l* ∼ 37.5 km, fringes superimposed on the beat spectra were visible. This suggests that the DFB laser coherence length was at least *l_coh_* ∼ 38 km with a corresponding linewidth, *Δλ_w_* ≤ 2.55 kHz. Within the experimental error determined by the optical delay length, the MI interferometer exhibits a similar vanishing of interference at a similar delay length, confirming the approximate coherence length value determined previously.

### 2.3. Voigt Fit

The measured beat spectra from the DSHI technique contains linewidth broadening contributions from both white and colored (1/f) noise sources which give rise to lineshape contributions that can be fitted with Lorentzian and Gaussian profiles, respectively. The Voigt function, a convolution of these two contributions, can be fitted to the beat spectra to separate out these components to the laser linewidth. This interpretation of noise dominated broadening is possible if the measured broadened linewidth is significantly larger than the natural laser linewidth (which should be Lorentzian). The Voigt profile is described by:

(1) $$V=\frac{2\;\text{ln}\;2}{\pi^{3/2}}\frac{w_{L}}{w_{G}}\cdot \int_{-\infty }^{+\infty }\frac{e^{-t^{2}}}{{\left(\sqrt{\text{ln}\;2}\frac{w_{L}}{w_{G}}\right)}^{2}+{\left(\sqrt{4\;\text{ln}\;2}\frac{f-f_{0}}{w_{G}}-t\right)}^{2}}\mathrm{dt}$$

where, *w_L_* and *w_G_* represent the full-width-half-maximum (FWHM) of the Lorentzian and Gaussian lineshapes and *f* is the frequency with *f_0_* being the centre frequency. The extracted Lorentzian linewidth must be divided by a factor of 2, and the Gaussian by a factor of $\sqrt{2}$ since the beat signal is a Voigt profile of two separate but identical spectral densities [13].

## 3. Results and Discussion

**Figure 3.**
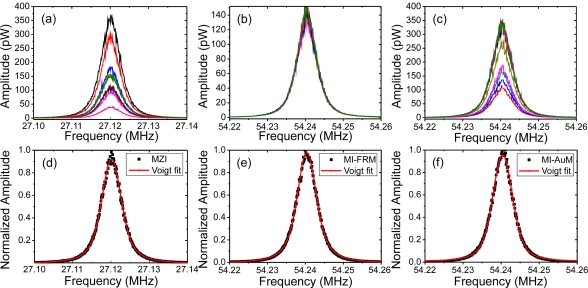
Beat spectra of 10 consecutive measurements each averaged 100 times for (**a**) MZI; (**b**) MI-FRM; and (**c**) MI-AuM at a DFB laser power of P = 116 μW. Voigt fitting for one of 10 acquired beat spectra for the DFB power of 116 μW for the (**d**) MZI; (**e**) MI-FRM; and (**f**) MI-AuM.

The beat spectra were recorded for six power levels of the DFB laser with each measurement averaged 100 times, taking ∼7 min to complete. A total of 10 measurements were taken within a minute of each other at each power level for both interferometers to reduce uncertainty and ensure statistical reliability and reproducibility. The FRMs of the MI were replaced with gold-coated mirrors (AuM) and the measurements were repeated for comparison. The beat spectra obtained for the resolution bandwidth (RBW) setting of 10 Hz are shown in Figure 3(a–c) for the MZI and MI-FRM and MI-AuM, respectively. Only three out of a total of 18 sets acquired are shown for the sake of clarity. The amplitude variations in the beat spectra observed for the MZI and the MI-AuM (Figure 3(a,c)) is a direct result of random variations in the polarization state of the two arms with respect to each other during the course of the measurements. This was evident despite the MZI and the MI-AuM being inside a soundproof enclosure and placed on an actively vibration stabilized table and the polarization state adjusted at the beginning of the first measurement (for each power level). The use of FRMs in the MI compensates for variations in polarization and produces consistent and repeatable beat spectra, as seen in Figure 3(b).

**Figure 4.**
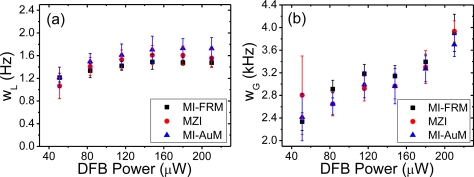
(**a**) Lorentzian, w_L_ and (**b**) Gaussian, w_G_ linewidths extracted through Voigt fitting of each measurement taken for the MZI and MI.

The data obtained for each of the spectra was fitted with the Voigt function; Figure 3(d–f) show one such case each for the MZI, MI-FRM and the MI-AuM. There is an excellent fit for the beat spectra obtained for all interferometers. The extracted Lorentzian and Gaussian linewidths through fitting of the Voigt function to each beat spectrum are plotted in Figure 4(a,b) for varying DFB output power. The MZI, MI-FRM and MI-AuM record a maximum Lorentzian linewidth of w_L_ = 1.62 kHz and w_L_ = 1.48 kHz and w_L_ = 1.72 kHz respectively, with the MZI and MI-AuM suffering from a noticeably larger measurement error than the MI-FRM. This is quantified in Table 1, where the mean values of the 10 measurements taken and their standard deviations for each power level are provided. The Lorentzian linewidths rollover with a slight downward trend at higher power for both interferometers, supporting the Schawlow-Townes equation where an inverse relationship is expected; it does not increase as observed in [9].

The Gaussian linewidth, w_G_, on the other hand, continues to increase with increasing power with measurements for both interferometers suffering relatively large measurement errors over the full range. However, the measurement error in the MI-FRM is still lower than the MZI for all measurements, except for the last measurement for 210 μW DFB power. The steady increase in the Gaussian linewidth is attributed to thermal fluctuations inside the DFB caused by increasing pump power. The spectral mismatch of the pump laser used (λ = 976 nm) to the Er^3+^ absorption line could have further exacerbated the pump dependence of the frequency noise of the DFB laser. The larger measurement error in the extracted Gaussian linewidth is attributed to the fundamental thermal noise sources listed above and the random nature of such noises.

The placement of the DFB laser (not including the pump laser) outside the soundproof box and off the vibration isolation table resulted in an increase of the Gaussian component by a factor ∼2 while the Lorentzian linewidth remained unchanged (within error). This significant increase is primarily attributed to increased ambient temperature fluctuations as a result of air currents over acoustic/vibrational fluctuations.

The extracted Lorentzian and Gaussian linewidths between the two interferometers are comparable with the MI-FRM exhibiting a slightly lower Lorentzian and a slightly greater Gaussian linewidth over the measurement range. The MI-FRM exhibits a lower error in measurement particularly for the extracted Lorentzian linewidth even though the losses in the interferometer were greater than the MZI and the MI with gold mirrors. This is attributed to the consistent signal achieved as a result of high polarisation stability. The MI-FRM should thus be able to produce similar results to the MZI with far fewer averages, potentially speeding up the measurement of systems. Further, with greater output powers from the fibre laser, it is conceivable that the MZI noise increases with longer delay lines whilst that of the MI improves.

## 4. Conclusions

A comparison of the Mach-Zehnder and Michelson interferometers was made in the context of direct linewidth measurements using the delayed self-heterodyne technique. The Michelson interferometer (MI) was configured in two ways: with Faraday mirrors (MI-FRM) and with standard gold mirrors (MI-AuM) to confirm the reduction in polarisation sensitivity using the FRMs and to demonstrate any advantages. The fitting of Voigt profiles to the acquired beat spectra for varying power levels of the DFB laser resulted in the extraction of comparable linewidths amongst all three interferometers, with the MI-FRM exhibiting the smallest measurement errors compared to the MZI and the MI-AuM. The beat spectra recorded for the MI-FRM is not susceptible to amplitude changes from random polarization drifts, providing the most accurate representation of the linewidth. Given the lower measurement error, the MI-FRM should be able to perform faster measurements of laser linewidths since a lower number of averages than either the MZI or MI-AuM can be used.

## Figures and Tables

**Table 1.** Extracted linewidths and standard deviations of measured linewidths for MI_FRM, MZI and MI-AuM.

	**Gaussian, *W_G_* (Hz)**						**Lorentzian, *W_L_* (Hz)**					

**DFB Power (μW)**	**MI-FRM**	**St. dev**	**MZI**	**St. dev**	**MI-AuM**	**St. dev**	**MI-FRM**	**St. dev**	**MZI**	**St. dev**	**MI-AuM**	**St. dev**

51	2,336	156	2,804	692	2,415	419	1,213	77	1,066	224	1,214	182
83	2,912	155	2,647	209	2,651	193	1,335	123	1,409	156	1,494	142
116	3,182	167	2,922	221	2,989	232	1,419	76	1,530	170	1,610	194
148	3,141	186	2,966	229	2,963	311	1,489	130	1,608	169	1,704	239
180	3,393	136	3,301	291	3,271	230	1,475	45	1,605	166	1,730	171
210	3,907	329	3,933	187	3,699	211	1,476	82	1,556	149	1,725	193
